# Case Report: Functional Analysis and Neuropsychological Evaluation of Dyshormonogenetic Fetal Goiter in Siblings Caused by Novel Compound Hyterozygous TPO Gene Mutations

**DOI:** 10.3389/fendo.2021.671659

**Published:** 2021-06-18

**Authors:** Tania Maria Barreto Rodrigues, Marlon Messias da Conceição Silva, Magali Maciel Freitas, Zélia Maria Costa Duarte, Vitória Sousa Frutuoso, Mariana Teixeira Rodrigues, Ileana Gabriela Sanchez Rubio

**Affiliations:** ^1^ Federal University of Juiz de Fora, Campus Governador Valadares, Governador Valadares, Brazil; ^2^ Thyroid Molecular Science Laboratory, Department of Biological Sciences, Federal University of São Paulo, Federal University of São Paulo (UNIFESP), Diadema, Brazil; ^3^ Structural and Functional Biology Program, Federal University of São Paulo, UNIFESP, São Paulo, Brazil

**Keywords:** fetal goiter, congenital hypothyroidism, thyroid peroxidase, mutations, neuropsychological evaluation, growth

## Abstract

**Introduction:**

It is rare for a euthyroid mother to carry a child with a fetal goiter. However, cases of congenital hypothyroidism (CH) caused by thyroid dyshormonogenesis have been reported. Even though gene mutations associated with fetal goiter have been reported in a few studies, the effects on intellectual development have not been investigated. This study aimed to characterize and investigate the underlying genetic mechanism of CH and neuropsychological development and growth of two siblings with CH-induced fetal goiters.

**Case report:**

Two male siblings from a non-consanguineous marriage with CH and fetal goiter were diagnosed by ultrasonography at 32- and 26-weeks of gestation. This condition was confirmed by cordocentesis in the first pregnancy (TSH: 135 μIU/ml). The mother was euthyroid, and no intra-amniotic levothyroxine treatment was performed. Peripheral blood DNA was screened for TPO mutations. The new deletion p.Cys296Alafs*21 and the p.Arg665Trp mutation, inherited from heterozygous parents, were identified in both patients. Functional analysis showed both mutations reduced the TPO enzyme activity and impaired the membrane localization. The p.Cys296Alafs*21 mutation produces a protein product with a drastically reduced molecular weight. Additionally, a complete clinical and neuropsychological evaluation was also performed. The WISC IV test was employed to provide an overall measure of the siblings’ cognitive and intellectual abilities. No growth retardation was detected in either child. In general, both children showed normal neuropsychological development; however, they exhibited slight reduction of Processing Speed Index scores, which are sensitive to neurological and attentional factors and motor maturation activity. Notably, the younger sibling obtained significantly low scores in the Operational Memory Index, a measure of attention capacity and psychoneurological immaturity.

**Conclusion:**

We described a new TPO compound heterozygosity that severely impaired the TPO activity and membrane localization leading to severe CH and fetal goiter. This is the first report showing the neuropsychological evaluation in patients with dyshormonogenetic fetal goiter. More studies are needed to understand the neurodevelopmental outcomes of neonates with CH-induced fetal goiters.

## Introduction

Congenital hypothyroidism (CH) is the most prevalent (1:2,000–1:4,000 live births) childhood endocrine disease and the most common cause of preventable intellectual disability (1). Most CH patients (80–85%) exhibit thyroid dysgenesis, which encompasses a spectrum of abnormalities, including agenesis (a complete absence of the thyroid gland), hypoplasia (a small gland with a typical location), and ectopy (an abnormally located gland) (2). In 10–15% of the cases, CH is caused by defects in the thyroid hormone biosynthesis or dyshormonogenesis (3). Mutations in thyroid peroxidase (TPO) (4), thyroglobulin (TG) (5), dual oxidase DUOX 1, DUOX maturation factor 1 (DUOXA1) (6), dual oxidase 2 (DUOX2) (7), DUOX maturation factor 2 (DUOXA2) (8), sodium iodide symporter (NIS) (9), and pendrin (SLC26A4) (10) have been associated with dyshormonogenesis.

Secondary to dyshormonogenesis, fetal goiters can also develop. These goiters are rarely large enough to be clinically detected at birth and even more challenging during intrauterine observation. However, advances in ultrasound technology have led to identifying many cases and may suggest fetal thyroid deficiency and hypothyroidism through peripheral gland hypervascularization and paradoxical increased fetal movement (11). It is important to point out that fetal goitrous hypothyroidism can also be caused by iodine deficiency (12), massive iodide exposure (13), and maternal hyperthyroidism therapy (14).

Up to now, mutations in TG, TPO, NIS, and DUOXA2 have been identified in few dyshormonogenetic fetal goiter patients (15–28) as shown in **Supplementary Table 1**. These mutations are usually transmitted in an autosomal recessive fashion; however, some studies have reported monoallelic mutations (29).

The effects of thyroid hormone and its deficit on children’s psychomotor and neurological outcomes have been well-documented (30), but not in CH-induced fetal goiter patients. Before fetal thyroid hormone synthesis begins at approximately 11 weeks of gestation (WG), the fetus’s brain is protected by the maternal thyroid hormone (31). After that point, fetal thyroid hormones are necessary for healthy development. A previous study screening neonates with CH caused by a total organification defect showed that their thyroid hormone levels were about 40–60% of normal levels and likely of maternal origin (32).

This study aimed to characterize and investigate the underlying molecular genetic mechanism for CH development and neuropsychological progression and growth of two siblings with CH-induced fetal goiters.

## Case Report

### Clinical Presentation

The present study follows two pregnancies of an initially 32-year-old primigravida. During the first pregnancy, a routine ultrasound scan (USS) at 28 WG did not identify any polyhydramnios or other fetal abnormalities. However, the USS at 32 weeks detected a fetal goiter (**Supplementary Figure 1**). The mother was subsequently referred for endocrinological evaluation. The mother stated that she does not take medication and that she presented thyroid hyperplasia two years earlier. As shown in **Table 1**, subsequent cordocentesis confirmed fetal hypothyroidism (TSH: 135 μIU/mL; FT4: 0.57 ng/dL). At 34 WG, the fetus was delivered by cesarean without any complications due to spontaneous premature labor. The male newborn weighed 2.145 kg and had Apgar scores of ten at 1 and 5 min. The newborn presented weak suction and vomiting and remained in the intensive care unit for five days. Serum thyroid hormones were measured two days after birth, and hypothyroidism was confirmed. The neonate was subsequently treated with levothyroxine (LT4).

**Table 1:** Thyroid hormone status of Patients 1 and 2 and of their parents and LT4 dose over time.

	TSH μIU/mL (0.3–5.0)	FT4 ng/dL (0.8–2.0)	TG ng/mL (1.35–35)	anti-TPO IU/mL (<9)	LT4 µg/day
**Patient 1: Fetal goiter detected at 32 WG; birth at 34 WG**					
32 WG Cordocentesis	135.0	0.57	–	3	–
At birth*	–	0.94	–	<10	–
2 days after birth	28.36	1.07	–	<10	25
2 weeks after birth	3.11	1.44	26.59	–	25
7 months	24.41	0.925			50
1.1 years old	2.02	1.71			50
1.9 years old	1.33	2.22	–	–	50
2.4 years old	8.68	1.68			75
3.2 years old	0.35	2.47	–	–	75
5 years old	2.52	1.94	–	–	62.5
10 years old	3.52	1.42	–	–	88
14.9 years old	6.69	1.13	–	–	150
15.8 years old	1.18	1.29	–	–	150
16.6 years old	6.98	1.36	–	–	150
**Patient 2: Fetal goiter detected at 26 WG; birth at 36 WG**					
2 days after birth	83.89	1.02	–	–	25
4 days after birth	12.68	1.70	530.6#	–	25
3 months after birth	2.91	1.88	–	–	25
6 months after birth	2.54	1.78	–	–	25
9 months after birth	11.49	1.29			50
1 year old	0.02	2.51	–	–	37.5
5.2 years old	9.57	1.28	–	–	62.5
10 years old	8.80	1.06	–	–	75
10.3 years old	1.64	1.48	–	–	75
11 years old	1.41	1.18	–	–	75
11.8 years old	2.65	1.33	–	–	75
**Mother** ^&^	1.99	0.97	–	5	N
**Mother** ^#^	0.93	1.10	7.91	1	N
**Father**	1.24	1.05	12.59	3	N

WG, weeks of gestation. * Not enough material for TSH testing; N, not in use; ^&^ in the third trimester of the first pregnancy with anti Tg:1 IU/mL (ref <40). ^#^ after the second pregnancy.

During the second pregnancy, the USS at 22 WG revealed no abnormalities. Like the first pregnancy, four weeks later (26 weeks), a fetal goiter was detected (**Supplementary Figure 1**). It was decided that no intervention would be administered at the time. Due to the progressive increase of the goiter volume, delivery was induced at 35 WG. The male child was delivered without complication and weighed 2.255 kg presenting Apgar scores of nine at 1 and 5 min. Two days after birth, congenital hypothyroidism was confirmed (TSH: 83.89 μIU/mL), and LT4 treatment was initiated. Thyroid hormone status of the patients and parents and LT4 doses over time are presented in **Table 1**.

Both siblings and the family showed good adherence to LT4 treatment; however, the patients still have goiter detected by USS. They showed good clinical conditions and puberty stages according to Tanner (33). Considering the height and body mass indices, no growth retardation was observed according to World Health Organization (5–19 years old) (34) (**Supplemental Figure 2**), and there were no motor problems detected in either child during the medical follow-up. Currently, Patient 1 is 16 years old and in his second year of high school. He has a height of 175 cm and weight of 55.7 kg, Height/Age and BMI/Age z scores of 0.08 and −1.19, respectively, and bone age of 17 years. He takes 150 mcg of levothyroxine per day. Patient 2 is 11 years old. He is in elementary school. He is 148.5 cm tall and weighs 39 kg. His calculated z scores for Height/Age and BMI/Age are 0.17 and 0.16, respectively, and his bone age is 10 years. Patient 2 is also treated with levothyroxine (75 mcg/day). In May 2021, laboratory thyroid tests of the siblings’ serum were TSH 6.98 µcIU/mL and 2.65 µcIU/mL (0.27–5.00) and FT4 (1.36 ng/dL and 1.33 ng/dL (0.75–2.00) for Patients 1 and 2, respectively. In Patient 1, a slight increase in TSH level was observed, with normal levels of FT4 (**Table 1**). It was then decided to maintain the levothyroxine dose and to repeat the tests in two months.

### Mutation Detection and Functional Assessment

**Figure 1:**
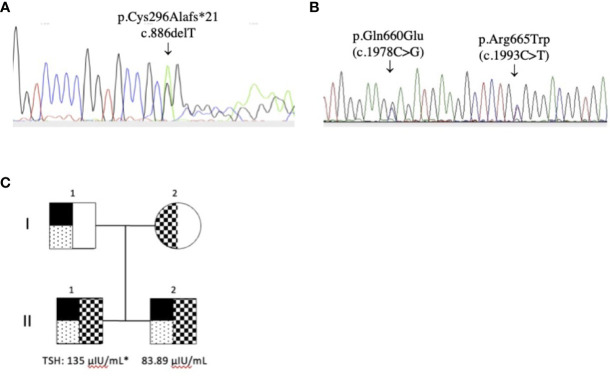
TPO gene mutations. **(A)** p.Cys296Alafs*21 (c.886delT) in exon 8 (reverse sequence) and **(B)** p.Gln660Glu (C.1978C>G) in exon 11 and p.Arg665Trp (c.1993C>T) in exon 11 (reverse sequence); **(C)** Pedigrees of the family and THS values at diagnosis (* cordocentesis). The p.Cys296Alafs*21 (c.886delT) and the p.Gln660Glu (C.1978C>G) mutation was detected in both patients (II.1 and II.2) and the father (I.1). The p.Arg665Trp (c.1993C>T) mutation was detected in both patients and the mother (I.2).

The sequences analyses revealed that the genomic DNA of the two siblings contained three TPO mutations: p.Gln660Glu (C.1978C>G) and p.Arg665Trp (c.1993C>T) in exon 11 and a new deletion p.Cys296Alafs*21 (c.886delT) in exon 8. Thep.Arg665Trp mutation was present in the mother’s DNA, and the p.Gln660Glu (4) and p.Cys296Alafs*21 (35) mutations were found in the father’s DNA (**Figure 1**). The p.Gln660Glu mutation is on the same allele as the deletion mutation; thus, it is unlikely to contribute to the disease. Thus, the presence of both the p.Arg665Trp and the p.Cys296Alafs*21 mutations indicates compound heterozygosity in the siblings’ DNA.

**Figure 2:**
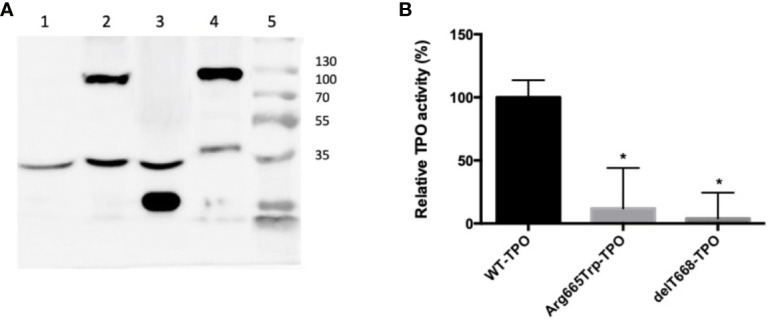
**(A)** Western blot of HEK293 extracts from cells transfected with wild-type or mutant TPOs. Lane: 1) pCDNA 3.1; Lane 2) Arg665Trp-TPO, 103 kDa; Lane 3) delT668-TPO, 35 kDa; Lane 4) TPO-WT; 103 kDa; Lane 5) molecular weight marker Thermo Scientific Page Ruler (Thermo Scientific, Carlsbad, CA). All strains expressed the endogenous control alpha-tubulin (53 kDa). **(B)** Extracellular enzymatic activity of HEK293 cells expressing wild-type or mutant TPOs. Enzymatic activity was assessed using the Amplex UltraRed reagent. *λ*_EXCITATION_ = 530 nm and *λ*_EMISSION_ = 560 nm. The values are expressed as a percentage of the TPO-WT transfected cells’ activity (*p < 0.05).

The pathogenicity of the p.Arg665Trp (Arg665Trp-TPO) and p.Cys296Alafs*21 (delT886-TPO) mutations was evaluated in HEK293 cells. The delT886-TPO mutation alters the protein reading frame and introduces a new stop codon at residue 316, resulting in a 35 kDa protein product. In comparison, the Arg665Trp-TPO missense mutant and TPO-WT migrate at a molecular weight of 103 kDa (**Figure 2A**). We also measured the enzymatic activities of TPO-WT, Arg665Trp-TPO, and delT886-TPO. As shown in **Figure 2B**, the TPO activities of Arg665Trp-TPO and delT886-TPO are significantly lower than TPO-WT levels.

**Figure 3:**
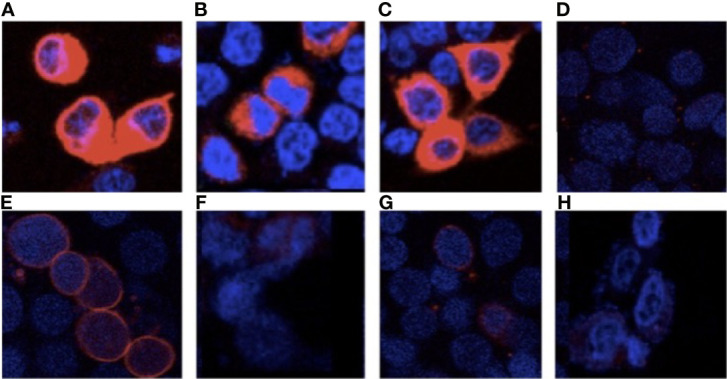
Cellular localization of wild-type and mutant TPOs. Immunofluorescence, using anti-TPO RPE5379- (Abcam) and AlexaFluor 594 (Invitrogen), under permeabilized conditions, Panel **(A)** TPO-WT, Panel **(B)** delT668-TPO, Panel **(C)** Arg665Trp-TPO, and **(D)** pcDNA and impermeabilized conditions Panel **(E)** TPO-WT, Panel **(F)** delT668-TPO, Panel **(G)** Arg665Trp-TPO, and Panel **(H)** pcDNA transfected HEK293 cells. Images were acquired with a Leica TCS SP8 confocal microscope at 63× magnification.

Next, we assessed TPO localization by immunofluorescence. Under cell permeabilizing conditions (**Figures 3A–D**), cytoplasmic TPO-specific immunostaining was observed in the wild-type and mutant TPO transfected cells. These results confirm TPO transfection and expression. Under non-permeabilizing conditions (**Figures 3E–H**), TPO-WT was localized along the cell membrane. Notably, we observed reduced membrane staining in cells transfected with delT886-TPO and Arg665Trp-TPO, indicating that these mutant proteins are not correctly inserted into the membrane (**Figures 3F, G**). It is also worth mentioning that no immunostaining was detected in cells transfected with empty pcDNA vector under either condition. The full methods are in the **Supplementary File**.

### Neuropsychological Evaluation

A clinical neuropsychologist evaluated the two brothers for attention (divided, sustained, and focused), cognitive flexibility, short- and long-term memory (verbal and visual), intellectual processes (reasoning, abstract thought, and critical thinking), motor functions (movements, laterality, and others), visual functions (perception, discrimination, and visuospatial and visuoconstructive organization), praxis, general intelligence, learning ability, and language (expressive, receptive and written). The children were also evaluated for the presence of Attention Deficit Hyperactivity Disorder (ADHD). Herein, the fourth edition of the Wechsler Intelligence Scale for Children (WISC IV) test to assess the siblings’ intelligence was used. These evaluations were performed at 11 years of age for Patient 1 and 6 years for Patient 2. A summary of these results is presented in **Table 2**. In general, the children exhibited behavior consistent with their age, sex, and education. Moreover, the evaluations showed that both boys had adequate age-related logical reasoning, conceptual verbal formation (abstract thinking), memory and verbal fluency, semantic knowledge, intellectual curiosity, and ability to deal with abstract symbols. Quantitative analyses of the WISC-IV results and comparisons of each patient with himself using the factorial indexes, which are considered more accurate measures of intelligence, showed that both patients exhibited lower Processing Speed Index (related to the speed of mental processing and motor graph), and motor visual organization scores. Additionally, Patient 2 had a significantly lower Operational Memory Index (OMI), according to the WISC-IV. The OMI provides a measure of the child’s attention. Due to its sensitivity, it has been utilized for detecting ADHD; however, to confirm the ADHD diagnosis, new evaluations are required between 7 and 12 years of age. Although Patient 2 is currently 11 years old, the parents refused consent for revaluation.

**Table 2:** Summary of the neuropsychological evaluation results of the dyshormonogenetic fetal goiter patients using WISC-IV Tests.

Characteristics	Patient 1	Patient 2
**Executive functions**		
Abstraction and judgment	Above average	Low Average
Sustained attention/concentration	Average	Low Average
Alternate attention	High average	Below average
Selective attention	Average	Low Average
**Verbal and non-verbal memory process**		
Non-verbal (visual) memory	Below average	High average
Verbal memory	Above average	High average
**Language**		
Verbal comprehension, naming, and vocabulary	Average	Average
Verbal fluency	Average	Average
**Constructive ability**		
Visual reproduction	Below average	–
Praxia	Low average	Low average
**Humor and behavior evaluation**		
Stress	Normal	Positive
Hyperactivity-TDAH	Negative	Hyperactivity and impulsivity*
**General intellectual operation (QI)**	Normal	Normal

Scores were categorized as well above average, above high average, average, below average, or well below average compared to their age reference group at each assessment. *To be confirmed in a future evaluation.

## Discussion

Fetal goiter is a rare thyroid disorder with an incidence of about 1:40,000 live births. Despite being a perinatal complication with low incidence, it can cause airway obstruction and polyhydramnios and increase the fetus’ risk of other morbidities and/or mortality (18, 36). This study investigated the genetic causes of CH and fetal goiter development and their impact on neurological functions. Herein, we identified mutations in the TPO gene of two brothers from a non-consanguineous marriage who exhibited fetal hypothyroidism and are currently undergoing LT4 treatment.

The high TSH and TG levels and the patients’ fetal goiters led us to investigate TPO gene mutations by sequencing patient DNA. Herein, we identified three mutations in both patients. These mutations include p.Gln660Glu (4) and p.Cys296Alafs*21 (a new deletion) inherited from the father, and p.Arg665Trp (35) inherited from the mother.

Studies have shown that TPO protein, an essential enzyme for thyroid hormone synthesis, is localized to the apical membrane of the thyrocyte (37). TPO activity depends on the proper conformation, cellular localization and an intact catalytic site (38). The new deletion, which introduces a stop codon at amino-acid 316 and produces a 35 kDa protein, exhibits significantly reduced TPO activity (**Figure 2**). The deletion results in the loss of the catalytic site (exons 8–10), the transmembrane domain (exon 15), and the extracellular domain (**Supplementary Figure 2**). The immunofluorescence experiments demonstrate that this mutant is localized in the cytoplasm and exhibits reduced membrane localization (**Figure 3**). It was previously reported that the TPO mutant p.Gln660Glu displays reduced activity and membrane localization (39). It is unlikely that the p.Gln660Glu mutation contributes to the disease since it is not present in the protein as it is located downstream of the truncating mutation p.Cys296Alafs*. Thus, our results indicate compound heterozygosity involving p.Cys296Alafs*2 and p.Arg665Trp, mutations present in the paternal and maternal alleles, respectively.

It should be pointed out that the p.Arg665Trp mutation, previously identified in two patients with severe dyshormonogenesis, one with a history of neonatal goiter, was found to have reduced activity in the guaiacol oxidation assays and no membrane localization (35, 40). These results are consistent with the present study’s data, showing that Arg665Trp-TPO exhibits drastically reduced enzymatic activity and plasma membrane localization when expressed in HEK-293 cells (**Figures 2** and **3**). In this sense, our results provide further evidence demonstrating the severity of the p.Arg665Trp mutation.

Fetal goiter is usually diagnosed during the second or third trimester (11), which coincides with the detection at 32 and 26 WG for Patients 1 and 2 in our study. This late manifestation is because the maternal thyroid hormone supports fetal development during the first trimester, with maternal T3 regulating neuronal proliferation and the onset of neuronal cerebral cortex migration (41). During the second trimester, the fetal thyroid hormones progressively contribute more to neurogenesis, neural migration, myelination, axonal growth, dendritic arborization, and glial differentiation. Finally, in the third trimester, maternal and fetal thyroid hormones are required for central nervous system maturation (42). It has been reported that maternal hypothyroidism negatively impacts the neuropsychological development of children (43), an outcome probably due to abnormal cortical development during the first trimester (44).

On the other hand, fetal hypothyroidism appears to affect cerebral areas later in development, influencing language, associative memory, auditory processing, attention, and executive processing (45). Additional evidence associating intrauterine brain damage with fetal hypothyroidism comes from children with severe CH due to agenesia or with a thyroid gland and very low neonatal FT4. These children face a higher risk for subtle irreversible neurological deficits despite early treatment after birth (46–48) and exhibit cognitive problems with memory, attention, and visuospatial processing that can persist into early adolescence (49). Furthermore, a summary of several studies demonstrated that children diagnosed with CH by neonatal screening and who received optimal therapy presented IQ reductions of about 0.5 SD (45).

In our study, the neuropsychological assessment indicated that in general these patients with fetal goiter exhibited a normal neuropsychological development, with some high or below average scores. For example, a deficit in working memory was identified in Patient 2. This type of deficit is typically related to the management of day-to-day information difficulties. Moreover, children with this deficit can have difficulties planning, ranking, establishing priorities, distinguishing importance, and engaging in activities requiring the manipulation of temporarily stored information. They usually need more time and put forth more effort to carry out tasks. In the school context, it is common to have difficulties in mathematics operations that involve multiple steps, remembering homework instructions and simultaneously processing multiple information sources (50). A decrease in the information processing index was observed in both patients, characterized by a slow visual motor function and diminishing perceptual-motor and attention functions. This index provides a measure of the ability to decode symbols and process new information. It is sensitive to neurological factors, motor maturation, and attentional factors. Thus, children with these deficits require more time to learn the same amount of information than children of their age and are more likely to be tired since additional effort is needed to perform tasks (50).

While it is easy to speculate that the siblings’ reduced neurological skills are related to fetal hypothyroidism, it would be premature to make this conclusion. Moreover, prenatal fetal goiter treatment has been proposed to reduce goiter volume, prevent comorbidities, and provide adequate thyroid status at birth, but this therapy remains controversial. A recent study confirmed the feasibility of a conservative intrauterine LT4 treatment; however, the medications (LT4 and/or T3), recommended doses, and administration methods have not been defined. The authors state that prenatal treatment risks and benefits must be considered case by case (29). Notably, a 2020**–**2021 consensus updated the guidelines for the diagnosis and management of congenital hypothyroidism (CH) (51). They strongly recommended intraamniotic T4 injections in a euthyroid pregnant woman with large fetal goiter related to hydramnios and/or tracheal occlusion.

Considering the long-term medical follow-up, both siblings exhibited good clinical conditions and normal growth. They showed good adherence to LT4 treatment and adequate hormonal status along with the increase of the LT4 dose. Previous studies have also shown normal growth development of fetal goiter patients under adequate treatment (15, 21).

In conclusion, this study discovered a novel compound heterozygous mutation, including a previously unknown deletion in the TPO gene. This mutation led to severe impairment in TPO activity and membrane localization and appeared to be associated with severe intrauterine CH and fetal goiter. Under adequate treatment, the long-term follow-up showed a normal growth progression of both patients. This is also the first report investigating the neuropsychological factors in CH-induced fetal goiter patients. More studies are needed to state the neurodevelopmental outcomes of dyshormonogenetic fetal goiter.

## Data Availability Statement

The datasets presented in this study can be found in online repositories. The names of the repository/repositories and accession number(s) can be found below: https://www.ncbi.nlm.nih.gov/genbank/, BankIt2432519 human MW660360.

## Ethics Statement

This study was approved by the UNIFESP Ethical Committee (CAAE 59240816.2.0000.5505). Written informed consent was obtained from a parent of the patients for the publication of any potentially identifiable images or data included in this article.

## Author Contributions

TR was the endocrinologist that diagnosed the fetal goiter in both patients and performed the follow-up. MS performed the mutagenesis and most of the immunofluorescence images. MF performed the DNA sequencing of the patient’s DNA. ZD performed the neuropsychological evaluation. VF performed the TPO activity evaluation. MR was responsible for the cell culture and transfection experiments. IR was the coordinator of the project. All authors contributed to the article and approved the submitted version.

## Funding

This work was supported by a grant from FAPESP 2014/24549-4, Sao Paulo State Research Foundation. This study was financed in part by the Coordenação de Aperfeiçoamento de Pessoal de Nível Superior – Brasil (CAPES) – Finance Code 001.

## Conflict of Interest

The authors declare that the research was conducted in the absence of any commercial or financial relationships that could be construed as a potential conflict of interest.
