# Supplementary material for: Case Report: Functional Analysis and Neuropsychological Evaluation of Dyshormonogenetic Fetal Goiter in Siblings Caused by Novel Compound Hyterozygous TPO Gene Mutations
Source: Front Endocrinol (Lausanne). 2021 Jun 18;12:671659. doi: 10.3389/fendo.2021.671659 (PMC8251258; doi:10.3389/fendo.2021.671659)
Supplement: Supplementary file 1 [file DataSheet_1.pdf]

Supplementary Material

Methods

Thyroid function tests

We determined the free T4, TSH, and TG serum concentrations using an electrochemiluminescence immunoassay (Roche Corporation, Indianapolis, IN). Thyroid volume was calculated using echographic studies, according to a previous study (52)

DNA sequencing

Identifying *TPO* mutations was achieved by isolating DNA from peripheral blood, amplifying the complete TPO sequence, as described previously (4), and sequencing the amplified products in an ABI 377 system (Applied Biosystems Corp., Foster City, CA). The resultant sequences were compared against the human *TPO* gene sequence (NM_207581). Herein, the A of the ATG start codon is referred to as +1, and the initiator methionine is termed codon 1. The parents and 100 healthy subjects without thyroid disease (serum FT4: 0.7–1.5 ng/dL and TSH: 0.5– 4.5 µIU/mL) were also screened for genetic mutations.

Mutagenesis

We introduced the identified mutations into wild-type TPO (TPO-WT), previously subcloned into pCDNA3.1, by site-directed mutagenesis using the Quick Change Lightning and XL II kits (Agilent Technologies, Santa Clara, CA). The following mutagenic primer pairs were used: c.886delT_F 5'-CGCTCTTCGGCCGCCGCGGCACCGGGGACC-3' and c.886delT_R 5'-GGTCCCCGGTGCCGCGGCGGCCGAAGAGCG-3' and Arg665Trp-F C>T 5'-CCAGTCACCGTCCCACAGAGCCTTCATCT-3' and Arg665Trp-R 5'-AGATGAAGGCTCTGTGGGACGGTGACTGG-3'.

Cell culture and transient transfections

In vitro experiments were conducted in HEK293 cells cultured in DMEM High Glucose media supplemented with 10% fetal bovine serum, 100 U/mL penicillin and 100 µg/mL streptomycin (Gibco, Gaithersburg, MD) at 37°C and 5% CO_2 atmosphere. HEK293 cells

(2×10^5) were aliquoted into six-well plates containing 13 mm round coverslips, cultured for 24 hours and then transfected with lipofectamine (Qiagen, Venlo, NE), 2 µg of TPO-WT or mutated TPO plasmids.

Western blotting

Seventy-two hours after transfection, protein extracts were collected, and total protein concentration was measured. Aliquots corresponding to 30 μg of total protein were separated using 8% SDS PAGE gels and transferred to nitrocellulose/PVDF membranes (Thermo Scientific, Carlsbad, CA) for subsequent immunoblotting. The membranes were incubated with a primary anti-TPO (1:5000, EPR 5379 Abcam, Cambridge, MA) and anti-α-tubulin antibody (1: 10000, SC-2004, Santa Cruz, Dallas, TX) antibodies. Signals were developed using Immobilon Western (Millipore, Burlington, MA), and immunoreactive bands were visualized with an ImageQuant LAS 4000 (GE Healthcare Life Sciences, Marlborough, MA).

TPO enzymatic activity

We used the Amplex UltraRed reagent (Invitrogen, Carlsbad, CA) to evaluate TPO enzymatic activity (53, 54). Briefly, transfected cells (1×10^6) were cultured in 6 well plates for 15 hours containing 20µM hemin (Sigma-Aldrich, St Louis, MO). Then 500 µL of reaction mixture containing 100 μM KI, 200 U/mL SOD (Sigma-Aldrich, St Louis, MO) and 50 µM Amplex UltraRed (Life Technologies, Carlsbad, CA) in PBS was added to each well. The reaction was initiated with 25 µL of H_2O_2. The plates were incubated in the dark for 10 minutes. An aliquot of 20 µL was then collected and mixed with 80 µL of stop solution containing 500 U/mL catalase (Sigma-Aldrich, St Louis, MO). and 100 U/mL of superoxide dismutase (Sigma-Aldrich). Fluorescence ($\lambda_{EXCITATION} = 530$ nm and $\lambda_{EMISSION} = 560$ nm) was measured in a Synergy HT Multi Detection Microplate Reader (BioTek Instruments, Inc. Winooski, VE). The mock-transfected control cells were considered zero. The TPO activities were calculated and expressed as a percentage of the TPO-WT transfected cells. Data are representative of two independent experiments performed in triplicate and was analyzed by Student's t-tests using the IBM SPSS Statistics software version 25.0. The level of significance was set to $p < 0.05$.

Indirect immunofluorescence

HEK293 cells were transfected on round coverslips, fixed in 4% paraformaldehyde for 20 minutes and blocked with 1% BSA in PBS and 0.1% triton for permeabilization condition. Then the cells were incubated with anti-TPO 1:100 (EPR 5379 Abcam, Cambridge, MA) for 1 hour in a humidity chamber in a blocking solution. Next, Alexa Fluor 594 (1:100) (Invitrogen, Carlsbad, CA) and DAPI (1:5000) (Invitrogen, Carlsbad, CA) were added and allowed to incubate for 1 hour in the dark. After mounting, the cells were visualized with a Leica TCS SP8 confocal microscope and analyzed using the LAS AF software.

Supplementary Table 1: Mutations identified in patients with CH and fetal goiter

Gene	Patients	Mutation	Reference
TG	One girl and One boy	c.1143delC; p.R2223H comp het	Caron et al.(15)
	Twin brothers	p.R1511X; p.G59S; p.S2113L comp het	Reynold et al. (16)
	One boy	p. R277X het as the euthyroid mother	Stoppa et al. (17)
	One boy	p.A1727Hfs*26p; p.G2375R het	Vasudevan et al. (18)
	One girl	p.E1835* het	Siffo et al. (19)
	One girl	c.5686+1delG hom	Stern et al. (20)
	Two brothers	p.Cys296Alafs*2; p.Arg665Trp; comp het	This study
	Two siblings	IVS30+1G>T	Rubio et al. (21)
TPO	One boy	p.Y453D; p.C800R comp het	Borgel et al. (22)
	One boy	p.W873X homo	Simm et al. (23)
	One boy	p.R89X het as the euthyroid father	Yapakçi et al.(24)
	One boy	c.1472G>A; c.1993C>T comp het	Figueiredo et al. (25)
	Two siblings	p.A397fs*76 het as the euthyroid father	Zdraveska et al. (26)
NIS	One boy	Comp het	Stoupa et al. (27)
DUOXA2	One boy	Comp het	Tanase-Nakao et al.(28)

Comp het: compound heterozygosity, hom: homozygosis, het: heterozygosis.

Supplementary Figure Captions/Legends

Supplementary Figure 1: Diagnosis of fetal goiter by ultrasound scan examination of (A) Patient 1 at 32 weeks of gestation and (B) Patient 2 at 26 weeks of gestation.

Supplementary Figure 2: Height and body mass indices of patients with CH-induced fetal goiters over time according to World Health Organization (5-19 years old) (34); (A) and (B): Patient 1; (C) and (D) Patient 2, respectively.

Supplementary Figure 3: TPO protein structure of (A) the wild type TPO with 933 amino acids and 103 kDa and (B) the mutant delT668-TPO (p.Cys296Ala fs*21) with 316 amino acids and 35 kDa. The localization of the p.Gln660Glu and p.Arg665Trp mutations are indicated. Adapted from Deladoey et al. (39)
