# Supplementary figures and images for: Case Report: Functional Analysis and Neuropsychological Evaluation of Dyshormonogenetic Fetal Goiter in Siblings Caused by Novel Compound Hyterozygous TPO Gene Mutations
Source: Front Endocrinol (Lausanne). 2021 Jun 18;12:671659. doi: 10.3389/fendo.2021.671659 (PMC8251258; doi:10.3389/fendo.2021.671659)

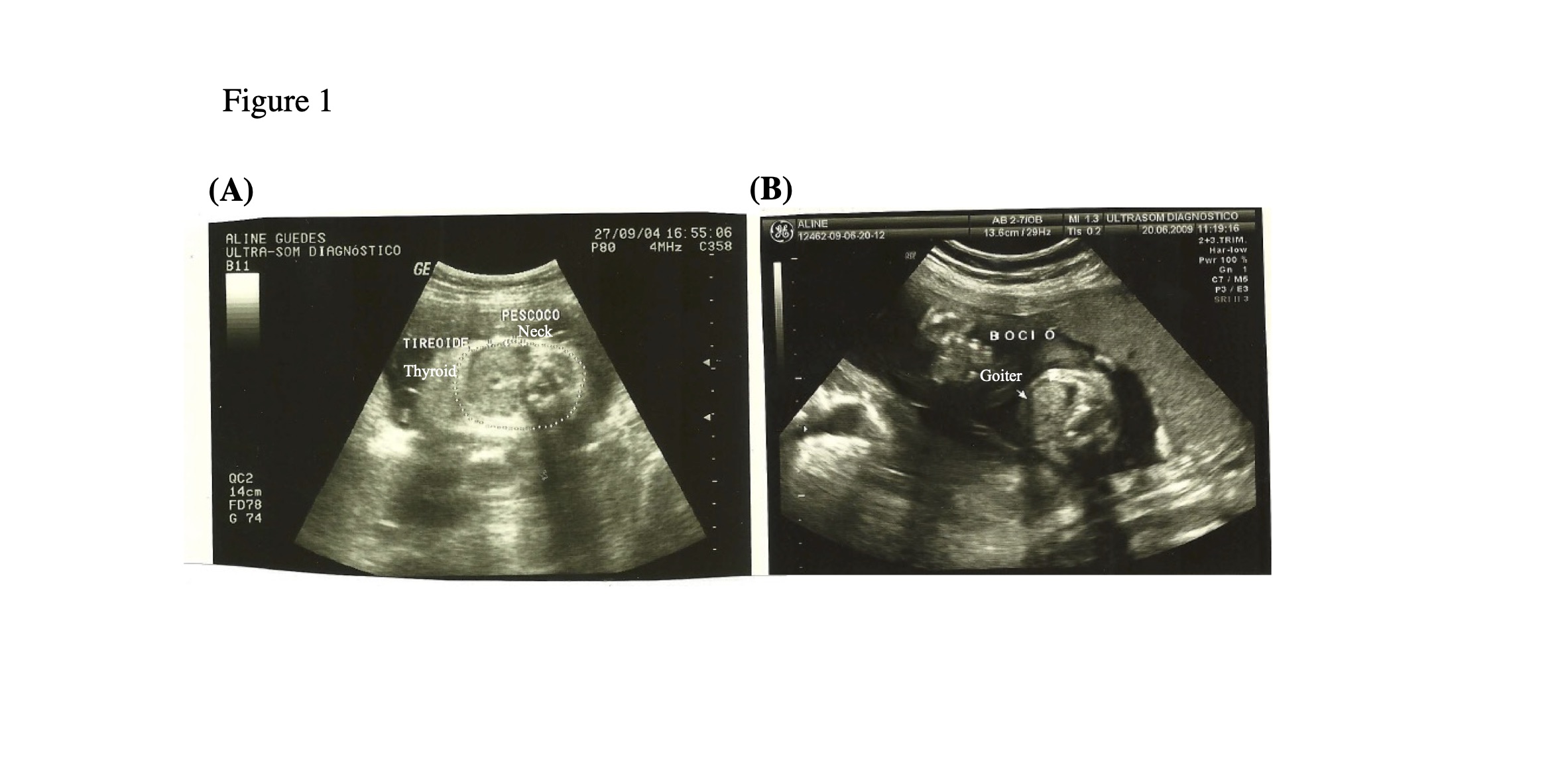

Supplement: Supplementary file 2 [file Image_1.jpeg]

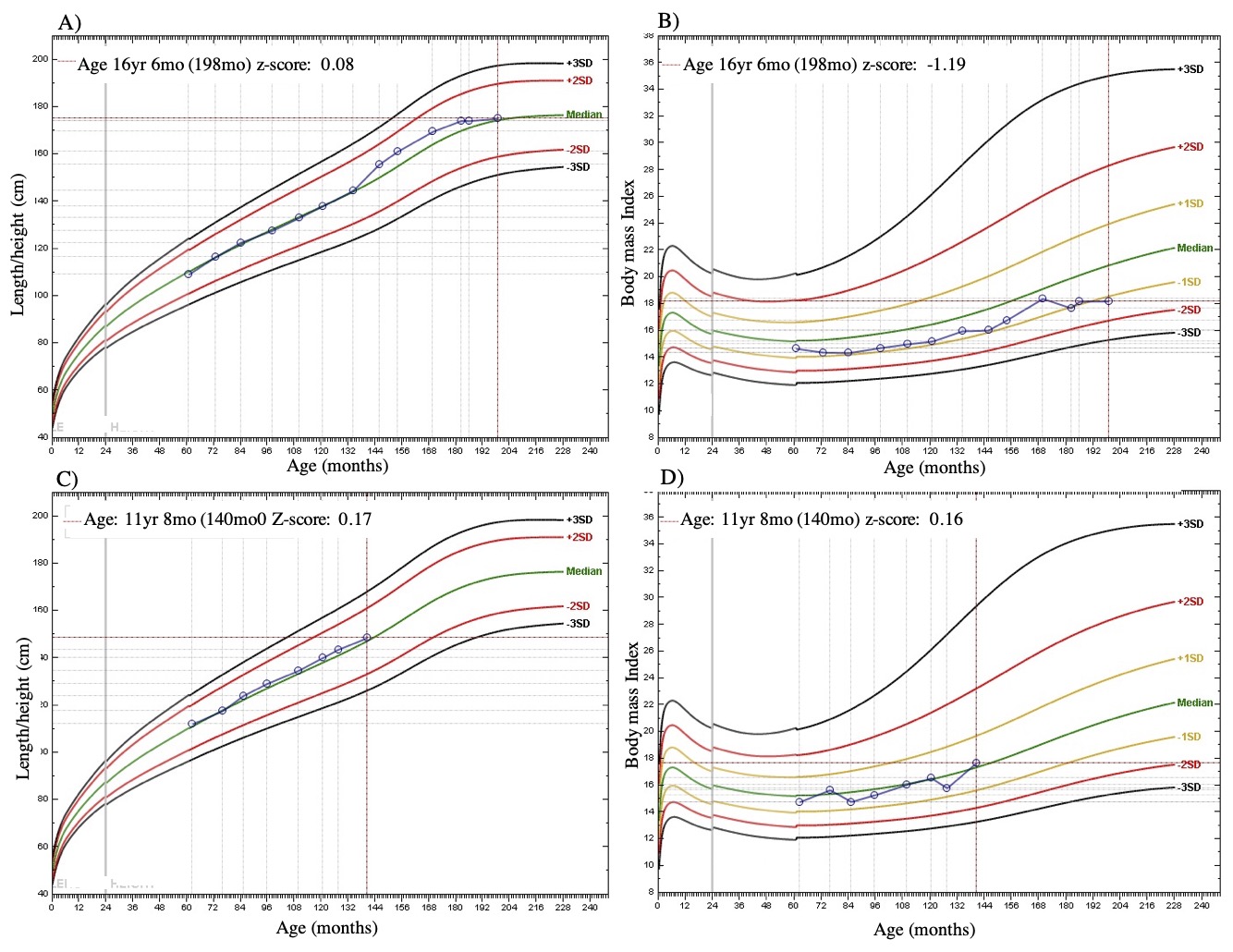

Supplement: Supplementary file 3 [file Image_2.jpeg]

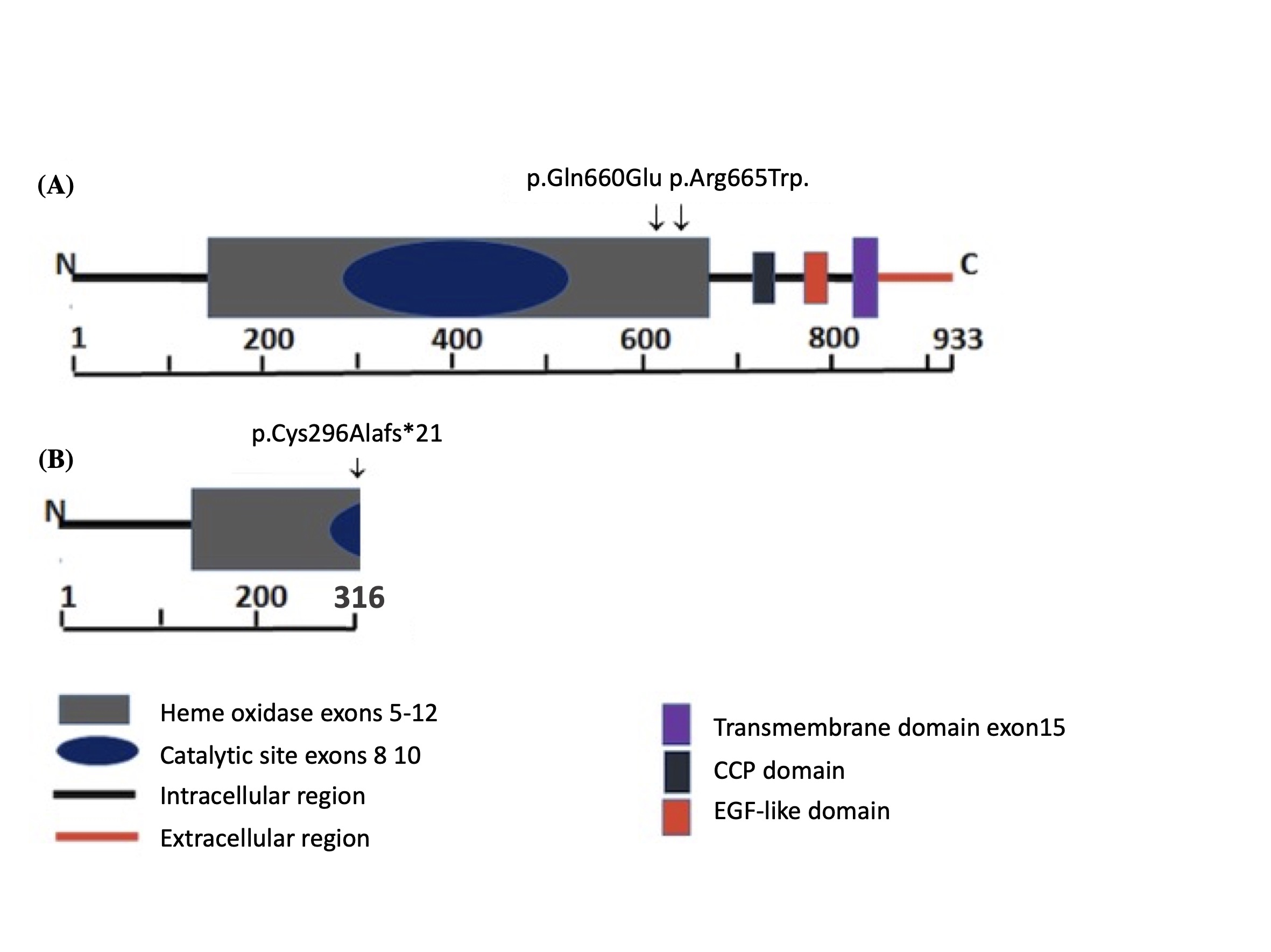

Supplement: Supplementary file 4 [file Image_3.jpg]
